# Government Intervention, Risk Perception, and the Adoption of Protective Action Recommendations: Evidence from the COVID-19 Prevention and Control Experience of China

**DOI:** 10.3390/ijerph17103387

**Published:** 2020-05-13

**Authors:** Taixiang Duan, Hechao Jiang, Xiangshu Deng, Qiongwen Zhang, Fang Wang

**Affiliations:** 1 Department of Sociology, Hohai University, Nanjing 211100, Jiangsu Province, China; duantaixiang@163.com; 2 Research Institute of Social Development, Southwestern University of Finance and Economics, Chengdu 611130, Sichuan Province, China; dxs@swufe.edu.cn (X.D.); ilxpw@swufe.edu.cn (Q.Z.); wf0911@smail.swufe.edu.cn (F.W.)

**Keywords:** COVID-19, government intervention, risk perception, adoption of PARs

## Abstract

This study examines the relationships between government interventions, risk perception, and the public’s adoption of protective action recommendations (PARs) during the COVID-19 coronavirus disease emergency in mainland China. We conducted quota sampling based on the proportion of the population in each province and gender ratios in the Sixth Census and obtained a sample size of 3837. Government intervention was divided into government communication, government prevention and control, and government rescue. We used multiple regression and a bootstrap mediation effect test to study the mechanism of these three forms of government intervention on the public’s adoption of PARs. The results show that government prevention and control and government rescue significantly increased the likelihood of the public adopting PARs. Risk perception was significantly associated with the public’s adoption of PARs. The effects of government interventions and risk perception on the public’s adoption of PARs was not found to vary by region. Risk perception is identified as an important mediating factor between government intervention and the public’s adoption of PARs. These results indicate that increasing the public’s risk perception is an effective strategy for governments seeking to encourage the public to adopt PARs during the COVID-19 pandemic.

## 1. Introduction

The World Health Organization has declared COVID-19 a pandemic after the disease, which was first detected in China in December 2019, rapidly spread to more than 150 countries. The control of the pandemic and its effects has become a focus of governments all over the world. Virologists are committed to research on the pathology of COVID-19, and to developing medical interventions. Social intervention is also playing an important role in control of the pandemic. Taking China as an example, the statistics for 24 March 2020 showed the number of active cases nationwide at 5166, with only 147 new cases from the preceding day. These numbers suggest that the epidemic has been effectively controlled in China. Unfortunately, few studies have focused on the role of government intervention in the prevention and control of epidemics and pandemics, with most previous studies concentrating on the public’s adjustments in their health-related behavior. By government intervention is meant the actions taken by the government to advise or mandate that the public and private sectors take certain measures to restrict the severity or spread of the effects of the pandemic. Furthermore, previous studies have lacked a discussion of the mechanism of government intervention on the public’s adoption of protective action recommendations (PARs) [1], and lacked an analysis of whether there are regional differences in the effects of government intervention on the public’s adoption of PARs. The aim of this study was to fill these gaps by investigating the relationships between government interventions, risk perception, and the public’s adoption of PARs.

Previous studies suggest that, in the absence of knowledge of a virus and effective drug interventions, it is necessary to rely on quarantine, restrictions on movement, and other behavioral measures that require cooperation to control the spread of epidemics in their early stages [2]. As a government’s effectiveness in responding to a public crisis is an important criterion for assessing whether it is a responsible government [1], the management of the epidemic in China has shifted from purely medical management to public crisis management. To prevent the spread of COVID-19, central, provincial, and municipal governments have successively initiated first-level responses to this major public health emergency, and implemented forceful intervention measures, such as mandated self-isolation at home. A recent study using a mathematical model to demonstrate the spread of COVID-19 confirmed that government interventions play a very important role in preventing and controlling the spread of the disease [3]. However, some studies have emphasized that the positive relationship between government intervention and the prevention and control of epidemics is based on the public’s active cooperation with government intervention [4,5]. Therefore, it is crucial to reveal how government interventions affect public’s adoption of PARs.

Other studies have argued that epidemic prevention and control requires more democratic measures, placing the emphasis on the adjustment of public protective behavior. Studies on health behavior adjustment are mainly based on the health belief model [5] and the protective behavior decision model [6]. The health belief model emphasizes the importance of risk perception in implementing protective action and deciding to adopt protective behavior, while the protective behavior decision model emphasizes the role of information sources in influencing the implementation of individual protective behavior through risk perception, stakeholder perception, and protective behavior perception. Many empirical studies based on these two models have assessed the public’s perception, response, expectations, and implementation of the protective behavior advocated by public health organizations [7,8]. These studies highlight the importance of subjectivity and risk perception in the implementation of protective behaviors, but neglect the interaction of social scenarios, institutions, cultural types, and other factors that influence the risk perception.

The present study attempts to integrate government intervention, risk perception, and the public’s adoption of PARs into a research framework. We make the following four contributions: First, we divide government intervention into three categories: government communication, government prevention and control, and government rescue, and analyze how these three categories of government intervention and risk perception affect the adoption of PARs. Second, we analyze the relationship between government intervention and risk perception. Third, we use multiple regression and a bootstrap mediation effect test to study the mechanism of government intervention on the public’s adoption of PARs. Existing studies have rarely considered mediation effects between government intervention and the public’s adoption of PARs. Fourth, we analyze regional differences in the effects of government intervention and risk perception on the public’s adoption of PARs.

## 2. Literature Review

### 2.1. Epidemic Management from the Perspective of Social Intervention

Some empirical studies by Chinese scholars have paid close attention to the government’s role in emergency management during public health emergencies, because the government has primary responsibility for governance in these circumstances. Indeed, since the emergence of COVID-19, the early warning, monitoring, and response to the crisis have been inseparable from the action of government [9]. Related research can be divided into two main topics: first, assessing the relationship between government intervention and the spread of epidemics from the perspective of epidemic prevention and control; and second, reflecting on the government’s experience in epidemic management from the perspective of public crisis governance of government.

Some studies have found government intervention to be effective in preventing and controlling the social transmission of epidemics [3,4,10]. Svoboda, Henry, Shulman et al. evaluated the spread of SARS in different environments in Toronto during the SARS epidemic, and found that after public health control measures were taken, the degree of transmission decreased significantly in non-controlled and non-residential environments and communities [10]. Chinese researchers used big data to trace back the spread of COVID-19 across the country, and its coefficient of infection, and demonstrated the Chinese government’s strong ability to control the spread of the epidemic and its positive contribution to preventing the spread both at home and abroad [3]. This indicates that China’s existing control measures have been effective. Similarly, predictive studies on the diffusion of epidemics have shown government intervention to be effective [11,12].

Nonetheless, there is evidence that government intervention is not always effective. Researchers who studied the effectiveness of the government’s prevention and control practices during the 2013 H7N9 epidemic divided the government’s intervention into three categories: government treatment, prevention and control, and government publicity and education. They found that some government control measures had a negative effect on the spread of the epidemic: for example, the release of a ‘prevention plan for the prevention and control of H7N9 of traditional Chinese medicine’, and ‘the launch of a daily reporting system for epidemic information’ produced negative social effects, such as public panic, and led to an increased cost of the government’s cooperation with the public. Therefore, the effectiveness of government prevention and control measures depends to a certain extent on the cooperation of the public [4]. The public’s willingness to participate and comply with the government’s prevention and control measures, and to enhance their knowledge of an epidemic and take the initiative to adjust their own behavior, are key to the success of government prevention and control measures aiming to block an epidemic. These empirical studies have shown the necessity of collaboration between the public and government but have not revealed how government interventions influence the public to produce cooperative behavior.

There is a long history of governmental responses to and prevention of epidemic diseases, such as the British government’s intervention on rinderpest, the prevention and control of epidemic diseases by the government in Beijing, and the prevention and the control of the cholera epidemic by the local government in Shandong province of China [11,12]. Zhang found that under crisis situations, such as the 2003 SARS epidemic, citizens’ behavior is subject to more direct constraints and restrictions than usual [1]. Under this circumstance, citizens must make necessary and urgent adjustments to their behavior, change many of their original habits, and cooperate with government prevention and control and intervention measures. This requires a benign interaction between the government and the public, emphasizing the responsibility of the government and the improvement of the government’s emergency management capabilities. Similarly, researchers in emergency management believe that in China’s political and social context, public participation follows an ‘external pressure model’ to promote policy agenda-setting and this is a key variable facilitating the transition from emergency management to public crisis governance [12,13]. To sustain a cooperative relationship between the government and the public, and for the public to better carry out the government’s prevention and control measures, the public’s need for the truth surrounding an epidemic crisis and the government’s responsibility to actually respond to the crisis must be met. Social intervention research in China on epidemic management has mainly highlighted the government’s responsibility and management capabilities rather than focusing on public participation.

Although some scholars have noticed the importance of the public’s cooperation, they have concentrated on the guidance of the government and the strengthening of the social mobilization ability of grassroots organizations of government to enhance the public’s participation in the government’s prevention and control work during public health emergencies [1]. The subjective role of public participation and cooperation has been less emphasized. In conclusion, research into epidemic governance has tended to ignore the empirical evaluation of the public’s subjective will when examining how to maximize the public’s voluntary participation in and adoption of PARs.

### 2.2. The Public’s Health Behavior Adjustments

There is a consensus within the academic community that the public’s adherence to government recommended protective behavior is crucial to the control of the spread of an epidemic [14,15,16]. Therefore, during an epidemic outbreak, the adjustment of public health behavior is an important topic for research.

The health belief model explains factors in the public’s adjustments to their health behavior during an epidemic outbreak, mainly taking account of susceptibility to the disease, the severity of the epidemic, the effectiveness of the protective actions, and the cost of taking the protective actions [5,17]. Gallagher et al.’s research into the protective behavior against skin cancer found that the adjustment of public health behavior was based on a high incidence of being affected and the knowledge held by individuals of the risk factors of their own behavior [18]. Other empirical studies based on the health belief models have examined the adjustment of health behavior during the SAR and H1N1 epidemics. These studies found that when individuals had a high level of perception of susceptibility to an epidemic, were convinced of the severity of the epidemic, and were sure that protective behavior was very effective, and perceived the cost of adopting protective behavior as lower, they were more likely to voluntarily adopt forms of protective behavior [19,20,21,22,23].

The health belief model emphasizes the psychological decision-making process behind individual protective behavior, with the formation of health beliefs regarded as the key to the adoption of protective behavior, but it does not consider how the public comes to accept the risk information that is provided on an epidemic and that promotes the generation of individual health beliefs. In proposing the protective action decision model, Lindell and Perry explained that the public’s attention to and understanding of the risk information disclosed in society and the circumstance affects risk perception, stakeholder perception, and protective behavior perception at the individual psychological level, and finally influences the adoption of protective behavior based on these perceptions and behavioral decisions [24]. Risk perception refers to an individual’s subjective evaluation of risk [25]. Protective behavior perception is an individual’s evaluation of the effective behavior in reducing risk [26]. Stakeholder perception is an individual’s evaluation of the professionalism, trust, and responsibility to protect the source of the risk information [24].

The related research shows that the health belief model emphasizes the importance of psychological factors such as risk perception, to protective behavior, while the protective action decision model explains how external information affects risk perception and, ultimately, individual adjustments of health behavior. Some studies have identified risk perception as a major contributing factor [23,27,28], while others have tested the moderating role of risk perception between protective behavior perception and stakeholder perception and the acceptance of protective measures recommended by public health departments. Lindell and Hwang, for example, studied risk perception as an intermediate variable [29]. Later, Wang studied how the public complied with protective behavior recommended by the government during the H7N9 epidemic, based on the two models, and found that risk perception, stakeholder perception, and protective behavior perception significantly affected publics’ compliance with PARs; using risk perception as an intermediary, Wang further showed that the effect of prevention and control measures advocated by government on an individual’s adoption of protective behavior was partially regulated by risk perception [30].

However, these studies have relied heavily on the effect of information sources on individual risk perception and neglected the effects on risk perception of the interaction of the information source with features of the social environment, such as social scenarios, institutions, and cultural types. Kasperson and colleagues proposed the social amplification of risk framework to explain the effect on the public’s perception of risk of the interaction between crisis events and individual psychology, social situations, institutions, and culture [31]. During the outbreak of COVID-19, the geospatial distribution of the spread of the disease has been intertwined with the Chinese government’s intervention policies, which provides social scenarios for the public’s perception of the risks of the disease.

From the sociologist Beck’s perspective, global society now takes the form of a risk society [32]. The government performs duties of epidemic governance and undertakes social intervention responsibilities against a social background combining global risk with the COVID-19 emergency crisis in the Chinese social scenario. The intervention policies that are formulated and the intervention measures that are implemented, such as government rescue efforts, government publicity, government prevention and control in different regions, involve direct relations with the public and make large changes to the social environment. According to the social amplification of risk framework, these measures will affect people’s risk perception, and then their decisions on whether to take protective actions. Based on the views presented above, we propose the following hypotheses:

**Hypothesis** **1.**  *Government intervention will significantly affect public protection behavior.*

**Hypothesis** **2.**  *Risk perception will significantly affect public protection behavior.*

**Hypothesis** **3.**  *Risk perception will be a mediating factor between government intervention and the public’s adoption of PARs.*

## 3. Methods

### 3.1. Participants and Procedure

The data for the present study came from a large-scale research project conducted by the Research Institute of Social Development of Southwestern University of Finance and Economics from 18–24 February in 2020, which investigated the psychosocial influence of COVID-19 in China. The project distributed questionnaires through the Internet and collected data anonymously. First, based on data representativeness and research funding constraints, we planned to conduct a nationwide survey with a sample size of 4000. Then, based on the population and sex ratio of each province reported in the Sixth Census, a quota design calculation was used to set a sample size for each province (Table A1). Finally, we distributed and collected the questionnaires through Wenjuanxing, an online survey system. Originally, 4096 people completed the questionnaire. As the purpose of the present study was to investigate the factors that influenced the public’s adoption of PARs in mainland China, the responses of 64 respondents from overseas were removed from the analyses. A further 195 respondents who did not meet quota requirements were also removed, leaving 3837 participants for analysis. 

### 3.2. Measures

Adoption of PARs: Four items from the Guidelines for the Public’s Protective Behavior for COVID-19, produced by the Chinese Center for Disease Control and Prevention [33], were adopted to measure the protective behaviors undertaken by the respondents (Table 1). For each of the recommended forms of protective behavior, the respondent was given a choice between complying and not complying. If the respondent had adopted all of the four recommended forms of protective behavior in the previous two weeks, he or she was considered to be a good adopter of the recommended protective behavior and assigned a value of 1. Other cases were assigned a value of 0.

Risk perception: Public conceptions of risk are complex and influenced by qualitative factors [34], including the extent to which a given risk is viewed as fatal, uncontrollable, and unknown. We adopted the measurement method of Liu et al. [35] and measured these factors using variables rated on 5-point Likert scales (Table 1). We conducted a factor analysis on these variables to generate a 3-item risk perception scale. The Cronbach’s alpha coefficient for the three items on this scale was 0.853, suggesting relatively high internal consistency. The response distribution was linearly transformed to range from 0 to 100, with 100 indicating the highest level of risk perception.

Government intervention: Government intervention was divided into government communication, government prevention and control, and government rescue (Table 1). Government communication refers to the communicative actions taken by the local government to promote public understanding of the COVID-19 pandemic, such as hanging banners and broadcasting. Government prevention and control refers to the mobilization of social forces by local governments to protect public health, such as the mobilization of medical workers to screen potential patients and of community workers to limit population movements. Government rescue refers to local government assistance and psychological support for COVID-19 patients, suspected patients, and other people. The Cronbach’s alpha values for government communication, government prevention and control, and government rescue were 0.865, 0.910, and 0.805, respectively, indicating good reliability.

Region: We divided the provinces of China into three regions: Eastern China, Middle China, and Western China. Middle China was made up of Hubei, Henan, Hunan, Anhui, Jiangxi, and Shanxi provinces, and Eastern China was made up of Hebei, Beijing, Tianjin, Shandong, Jiangsu, Shanghai, Zhejiang, Fujian, Guangdong, and Hainan provinces. The other provinces were assigned to Western China. For the promotion of regional economic development, the Chinese government divided the provinces of China into these three regions in 1986. As the other five middle provinces are adjacent to Hubei and are also the areas most affected by COVID-19, we adopted this division for our study.

We also controlled for the demographic characteristics of gender, age, household registration, years of schooling, marital status, and number of family members. The descriptive statistics for each variable are shown (Table 2).

### 3.3. Research Methods

To test the hypothesis that the relationship between government intervention and adoption of PARs is mediated by risk perception, we conducted mediation analysis using the four-step procedure of MacKinnon et al. [36]. Generally, mediation can be said to occur when the independent variable significantly affects the mediator, the independent variable significantly affects the dependent variable in the absence of the mediator, the mediator has a significant unique effect on the dependent variable, and the effect of the independent variable on the dependent variable shrinks upon the addition of the mediator to the model. Therefore, we followed these steps to test the regression coefficients of each model in turn.

As we were interested in whether the public fully complies with the recommended protective behaviors and the dependent variable was a dummy variable, we used a Logit regression model to examine the effects of government intervention on compliance with recommended protective behavior, the effects of risk perception on compliance with recommended protective behavior, and the effects of government intervention and risk perception on compliance with recommended protective behavior. To make the results easier to understand, we have reported the odds ratios (OR) of each independent variable on the dependent variable, rather than the coefficient. For the effect of government intervention on risk perception, as risk perception is a continuous variable, we used multiple linear regressions and the ordinary least squares (OLS) method for the estimate. 

As we used an online survey, some older populations would have been missed because of their lower rates of Internet use, and because the quota sampling was not a random process, our sample was biased towards overrepresenting the younger population. To address these sampling issues, we used bootstrap statistical inference. Bootstrapping allows inferences to be made with unknown sampling distributions for producing reliable confidence intervals [37].

The mediating effect of risk perception between government intervention and adoption of PARs was tested adopting the bootstrap estimation procedure in STATA 16 (Stata, College Station, TX, USA). Mackinnon et al. [36] suggested that the bootstrap method yields the most accurate confidence intervals for indirect effects. The reason for the bootstrapping approach is that the standard error estimates and confidence intervals of indirect effect will usually be imprecise because the indirect effect estimates generally do not follow a normal distribution [36]. The power of the statistical tests for the indirect effects which assumed to be normally distributed can be suspected. Thus, the bootstrap method can be a good option to analyze indirect effects [36,38]. The confidence intervals in this paper are all based on bootstrapping with 1000 iterations.

## 4. Results

### 4.1. Government Intervention, Risk Perception, and Adoption of Protective Action Recommendations

Correlational analyses in Table 3 shows that government communication, government prevention and control, and government rescue were positively related to risk perception and the public’s adoption of PARs. Risk perception was positively related to the public’s adoption of PARs. Furthermore, it was found that risk perception was also positively related to government communication, government prevention and control, and government rescue respectively. While the Chinese government’s social interventions, which range from government communication to government prevention and control to government rescue, have been very rigorous, the intensity of government intervention varies from region to region (Table A2). Therefore, in addition to examining the relationships between government interventions, risk perception, and the public’s adoption of PARs, we also examined regional differences in the effects of government intervention and risk intervention on the public’s adoption of PARs.

To estimate the influence of risk perception on the public’s adoption of PARs, and how this effect varies across regions, we use the same set of logit regression models for each outcome variable in Table 4. As the findings from Model 1 show, gender, age, marital status, number of family members, and region are associated with the public’s adoption of PARs. However, Model 2 shows that when we add the risk perception variable, only household registration, year of schooling, and region remain statistically significant. Model 2 also shows that risk perception is positively associated with the public’s adoption of PARs (OR = 1.025, 95% CI: 1.023 to 1.028). Controlling for individual, family, and regional characteristics, the higher the respondent’s score for risk perception, the greater the likelihood of adopting PARs. We can see that the pseudo *R*^2^ of the regression increased from 0.034 to 0.166, which suggests that risk perception is an important predictor of the public’s compliance with PARs.

Model 3 in Table 4 shows that when the interaction term between risk perception and region is added, risk perception is also positively associated with the public’s adoption of PARs (OR = 1.024, 95% CI: 1.020 to 1.028), while region has no significant effect on the public’s adoption of PARs. Meanwhile, the interaction term is not statistically significant, and there was no increase in pseudo *R*^2^. These results imply that our hypothesis 2 was supported by the data, and the effect of risk perception on the public’s adoption of PARs did not vary by region. 

To estimate the influence of government intervention on the public’s adoption of PARs, and how this effect varied across regions, we also used the same set of logit regression models for each outcome variable in Table 5. The results for Model 1 show that government interventions are related to the public’s adoption of PARs. Specifically, controlling for individual, family, and regional characteristics, government prevention and control is significantly associated with the public’s adoption of PARs (OR = 1.024, 95% CI: 1.018 to 1.031), government rescue is also significantly associated with the public’s adoption of PARs (OR = 1.009, 95% CI: 1.006 to 1.013), but government communication has no significant effect on the public’s adoption of PARs (OR = 1.004, 95% CI: 1.000 to 1.008). These results suggest that government interventions are important predictors of the public’s compliance with PARs.

Models 2–4 in Table 5 show that when the interaction term between government intervention and region is added, government communication has no significant effect on the public’s adoption of PARs, government prevention and control and government rescue still show a significant association with the public’s adoption of PARs, while region has no significant effect on the public’s adoption of PARs. Meanwhile, the interaction term is not statistically significant, and we found no significant increase in pseudo *R*^2^. These results imply that our hypothesis 1 is supported by the data and the effect of government intervention on the public compliance with recommended protective behavior does not vary by region.

Model 1 in Table 6 presents the OLS model results with risk perception as the dependent variable. The results show that government interventions have a significant association with risk perception. Specifically, controlling for individual, family, and regional characteristics, government communication has a significant association with risk perception (B = 0.090, 95% CI: 0.040 to 0.139), government prevention and control has a significant association with risk perception (B = 0.458, 95% CI: 0.374 to 0.542), and government rescue has a significant associated with risk perception (B = 0.190, 95% CI: 0.138 to 0.243). These results suggested that government interventions are important predictors of risk perception.

Model 2 in Table 6 presents the logit model results with the public’s adoption of PARs as the dependent variable. The results show that when the risk perception is included, government prevention and control and government rescue still have a significant impact on the public’s adoption of PARs, which is in line with Model 1 in Table 5. Compared with Model 1 in Table 5, the effect of government communication on the public’s adoption of PARs (OR = 1.014, 95% CI: 1.007 to 0.022), and the effect of government prevention and control on the public’s adoption of PARs (OR = 1.008, 95% CI: 1.004 to 1.014) all become significantly smaller. This result indicates that government intervention affects compliance with recommended protective behavior through risk perception, and that risk perception is an intermediary variable between government intervention and the public’s adoption of PARs.

### 4.2. Bootstrap Estimation of Mediation Effects

To further test the mediation effect of risk perception between government intervention and the public’s adoption of PARs, we used bootstrapping to analyze the mediating effects (Table 7). The results show, first, that government communication has no significant effect on the public’s adoption of PARs. Second, the results show that risk perception plays a mediating role between government prevention and control and the public’s adoption of PARs, with the mediating effect of risk perception being significant and having an indirect effect value of 0.075 (*p* < 0.001, 95% CI: 0.061 to 0.095). The intermediary effect accounts for 31.88% of the total effect. Finally, the results show that risk perception plays a mediating role between government rescue and the public’s adoption of PARs, with the mediating effect of risk perception being significant and having an indirect effect value of 0.043 (*p* < 0.001, 95% CI: 0.030 to 0.055). The mediating effect accounts for 34.68% of the total effect. Therefore, our hypothesis 3 is supported by the data.

## 5. Discussion

This study analyzed data from a large-scale research project that investigated the psychosocial influence of COVID-19 in mainland China to examine the relationships between government interventions, risk perception, and the public’s adoption of PARs. The health belief model and the protective action decision model emphasize the importance of risk perception on the public’s adoption of PARs [5,17], while the perspective of social intervention emphasizes the importance of government intervention on people adjusting their health behavior [3,4,10]. Against this background, the current study makes two important contributions to the literature. First, we analyzed the relationship between government intervention and risk perception and found that government interventions were associated with the public’s risk perception. Second, we systematically analyzed the effects of risk perception and government interventions on the public’s adoption of PARs. Multiple regression and a bootstrap mediation effect test showed government intervention to be significantly associated with the public’s adoption of PARs, and risk perception to be an important mediating factor between government intervention and the public’s adoption of PARs. The findings help us to produce a fuller picture of the determinants of the public’s adoption of PARs.

Four main points of discussion emerge from the results. First, the findings show that while government interventions increased the likelihood of the public’s adoption of PARs, not all three types of government intervention had a significant association with the public’s adoption of PARs. Specifically, two forms of intervention, government prevention and control and government rescue, significantly increased the likelihood of the public’s adoption of PARs, but government communication had no significant effect on the public’s adoption of PARs. These results are consistent with previous studies [3,10]. They imply that the government’s rapid response to public health emergencies not only allows for direct and timely treatment of patients, but also increases the likelihood of the public’s adoption of PARs, thereby reducing the social transmission of the virus. The results also have important practical implications. When a public health emergency occurs, the public may be unaware of the potential dangers because of poor information and will decide whether to take protective measures based on the government’s response. This requires the government to be able to scientifically assess the crisis level of the epidemic [32] and to guide the public in taking the correct protective measures.

Second, the findings show that risk perception had a significant association with the public’s adoption of PARs. Specifically, the higher a respondent’s score for risk perception, the greater the likelihood of adopting PARs. This result is consistent with previous studies [5,6,30], and indicates that improving the public’s perception of risk is a key factor in driving compliance with recommended forms of protective behavior. Unlike previous studies, risk perception in this study is a comprehensive concept that encompasses three aspects of risk to the public: the perceived severity of COVID-19, the mortality rate of COVID-19, and the extent to which COVID-19 affects their lives. Previous studies based on the health belief model have shown that individuals with a high level of perception of susceptibility to an epidemic and of its severity and those who were more certain that protective behavior was very effective, were more likely to voluntarily adopt protective behavior during the SAR and H1N1 epidemics [18,19,20,21,22]. It would be interesting to further assess the relative effect of different types of risk perception on the public’s adoption of PARs.

Third, the results show that the effects of government intervention on the public’s adoption of PARs did not vary by region, and that the effects of risk perception on the public’s adoption of PARs also did not vary by region. This indicates that, with an equal intensity of government intervention, there is no significant difference in the likelihood of the public’s adoption of PARs across regions. These findings did not match our expectations and may be due to two factors. First, the Chinese government has made very stringent interventions since the outbreak of COVID-19, which has greatly increased the likelihood of the public’s adoption of PARs. Second, COVID-19 shares many similarities with SARS, and the respondents’ personal experiences with the SARS epidemic, might have led them to easily perceive the severity of COVID-19, thus increasing their perception of risk and, in turn, the likelihood of the adoption of PARs.

Finally, the results show that risk perception is an important mediating factor between government intervention and the public’s adoption of PARs. In previous studies, the relationship between government intervention and the public’s adoption of PARs and the relationship between risk perception and public’s adoption of PARs have represented two different research pathways. A recent study showed that the effect of government prevention and control measures on an individual’s adoption of PARs was partially regulated by risk perception [28]. In this study, we further examined the relationships between government interventions, risk perception, and the public’s adoption of PARs, and found that although all three types of government intervention had a significant association with risk perception, only government prevention and control and government rescue influenced the likelihood of the public’s adoption of PARs through risk perception. These results imply that increasing the likelihood of the public’s adoption of PARs requires a range of strategies that go beyond merely telling people what to do.

These are several limitations to this study. First, with China in a critical period of epidemic prevention and control at the beginning of this project, we were not able to select respondents at random, which led us to adopt a quota sampling strategy rather than a random sampling strategy. The use of a quota sampling strategy meant that we could not use the traditional statistical inference methods to infer the actual patterns among the general population. Second, as we used an online survey method, the older population was underrepresented because of their lower rates of Internet use. To address this problem, we used bootstrapping for statistical inference. Third, government intervention and risk perception were based on self-reported measures, and respondents might have provided socially desirable for government intervention and risk perception. Fourth, when facing an emergency, the Chinese people are known for a strong spirit of collectivism and willingness to cooperate. The relationship between government intervention, risk perception, and the public’s adoption of PARs needs to be studied in other cultural contexts. Despite these limitations, the study brings important insights promoting our understanding of how government intervention influences the public’s adoption of PARs.

## 6. Conclusions

Three categories of government interventions, government communication, government prevention and control, and government rescue, showed different effects on the public’s adoption of PARs. Specifically, two forms of intervention, government prevention and control and government rescue were found to have significantly increased the likelihood of the public’s adoption of PARs. Risk perception was associated with the public’s adoption of PARs. Neither the effect of government interventions nor the effect of risk perception on the public’s adoption of PARs varied by region. Risk perception was identified as an important mediating factor between government intervention and the public’s adoption of PARs. The indirect effects of government prevention and control on the public’s adoption of PARs through risk perception accounted for 31.88% of the total effect. The indirect effects of government rescue on the public’s adoption of PARs through risk perception accounted for 34.68% of the total effect. These findings suggest that increasing the public’s risk perception is an effective strategy for governments seeking to encourage the public to adopt PARs during the COVID-19 pandemic.

## Figures and Tables

**Table 1:** Key variables and questionnaire items.

Variable	Question
Adoption of PARs (0 = no,1 = yes)	Have you taken the recommended protective action of self-isolation at home in the past 2 weeks? Have you taken the recommended protective action of wearing a mask when going out in the past 2 weeks? Have you taken the recommended protective action of covering your mouth with tissues or elbows when sneezing or coughing in the past 2 weeks? Have you taken the recommended protective action of washing your hands immediately on arriving home in the past 2 weeks?
Risk perception (1 = not at all seriously, 5 = very seriously)	How seriously do you take the COVID-19 epidemic in mainland China? How seriously do you take effect of COVID-19 risk on your life? How seriously do you take the risk of fatality from COVID-19?
Government communication (1 = not at all frequent, 5 = very frequent)	To what extent do you think your local government uses banners to raise public awareness of and recommend protective behavior against the 2019-nCoV? To what extent do you think your local government uses broadcast to raise public awareness of and recommend protective behavior against the 2019-nCoV? To what extent do you think your local government uses brochures to raise public awareness of and recommend protective behavior against the 2019-nCoV? To what extent do you think your local government uses WeChat or text messages to raise public awareness of and recommend protective behavior against the 2019-nCoV?
Government prevention and control (1 = not at all sufficient, 5 = sufficient)	To what extent do you think your local government mobilizes medical workers for the prevention and control of COVID-19? To what extent do you think your local government mobilizes community workers for the prevention and control of COVID-19? To what extent do you think your local government mobilizes social organizations for the prevention and control of COVID-19? To what extent do you think your local government mobilizes volunteers for the prevention and control of COVID-19? To what extent do you think your local government mobilizes property personnel for the prevention and control of COVID-19? To what extent do you think your local government mobilizes experts and scholars for the prevention and control of COVID-19?
Government rescue (1 = not at all sufficient, 5 = sufficient)	To what extent do you think your local government has designated hospitals to receive and treat patients with COVID-19? To what extent do you think your local government has specified hospitals for the medical observation of patients suspected to have COVID-19? To what extent do you think your local government has made a psychological hotline available for psychological counselling?

local government refers to the county government.

**Table 2:** Descriptive statistics for the main variables.

Variables	Mean	Std. Dev.	Freq.	%
Adoption of PARs				
Yes			3039	79.20
No			798	20.80
Risk perception	60.59	41.10		
Government communication	73.31	30.33		
Government prevention and control	72.96	22.25		
Government rescue	76.08	30.27		
Years of schooling	15.99	2.495		
Number of family members	3.876	1589		
Gender				
Male			1985	51.73
Female			1852	48.27
Age group (Years)				
<30			3063	79.83
30–60			704	18.95
>60			70	1.88
Household registration				
Rural household			1212	31.59
Urban household			2625	68.41
Marital status				
Unmarried			2136	55.67
Married			1701	44.33
Region				
Eastern China			1463	38.13
Middle China			1064	27.73
Western China			1310	34.14

**Table 3:** Correlations of government intervention, risk perception, and the public’s adoption of protective action recommendations (PARs).

Variables	1	2	3	4	5
1. Adoption of PARs	1				
2. Government communication	0.271 *	1			
3. Government prevention and control	0.358 *	0.686 *	1		
4. Government rescue	0.329 *	0.520 *	0.720 *	1	
5. Risk perception	0.402 *	0.351 *	0.451 *	0.416 *	1

* Correlation is significant at the 0.05 level (2-tailed).

**Table 4:** Odds ratios and 95% confidence intervals from logit models of the public’s adoption of protective action recommendations (PARs) on risk perception and sociodemographic characteristics.

Variables	Model 1	Model 2	Model 3
	Logit	Logit	Logit
Gender			
Male	Reference	Reference	Reference
Female	1.43 ***	1.047	1.041
	(1.218, 1.681)	(0.878, 1.249)	(0.873, 1.242)
Age group (years)			
<30	Reference	Reference	Reference
30–60	2.307 ***	1.289	1.292
	(1.703, 3.124)	(0.930, 1.786)	(0.931, 1.792)
>60	0.549 *	0.988	0.983
	(0.319, 0.943)	(0.555, 1.759)	(0.552, 1.749)
Household registration			
Rural household	Reference	Reference	Reference
Urban household	0.841	1.350 **	1.356 **
	(0.699, 1.012)	(1.098, 1.660)	(1.103, 1.668)
Years of schooling	0.970	0.946 **	0.948 **
	(0.936, 1.005)	(0.908, 0.985)	(0.910, 0.987)
Marital status			
Unmarried	Reference	Reference	Reference
Married	1.470 ***	1.077	1.071
	(1.210, 1.786)	(0.873, 1.329)	(0.868, 1.322)
Number of family members	0.948 *	1.024	1.024
	(0.901, 0.997)	(0.970, 1.081)	(0.970, 1.081)
Region			
Middle China	Reference	Reference	Reference
Western China	1.008	1.347 **	1.305
	(0.825, 1.232)	(1.082, 1.676)	(0.974, 1.747)
Eastern China	1.295 *	1.279 *	1.102
	(1.059, 1.582)	(1.029, 1.590)	(0.808, 1.504)
Risk perception		1.025 ***	1.024 ***
		(1.023, 1.028)	(1.020, 1.028)
Risk perception × Western China			1.000
			(0.995, 1.006)
Risk perception × Eastern China			1.004
			(0.998, 1.009)
*N*	3837	3837	3837
pseudo *R*^2^	0.034	0.166	0.166

95% CI in parentheses; * *p* < 0.05, ** *p* < 0.01, *** *p* < 0.001.

**Table 5:** Odds ratios and 95% confidence intervals from logit models of the public’s adoption of protective action recommendations (PARs) on government intervention and sociodemographic characteristics.

Variables	Model 1	Model 2	Model 3	Model 4
	Logit	Logit	Logit	Logit
Gender				
Male	Reference	Reference	Reference	Reference
Female	1.241 *	1.240 *	1.235 *	1.236 *
	(1.043, 1.476)	(1.042, 1.474)	(1.038, 1.469)	(1.039, 1.470)
Age group (years)				
<30	Reference	Reference	Reference	Reference
30–60	1.750 ***	1.761 ***	1.772 ***	1.755 ***
	(1.272, 2.407)	(1.279, 2.424)	(1.286, 2.441)	(1.275, 2.418)
>60	0.745	0.742	0.729	0.744
	(0.416, 1.335)	(0.415, 1.327)	(0.408, 1.303)	(0.415, 1.332)
Household registration				
Rural household	Reference	Reference	Reference	Reference
Urban household	1.091	1.090	1.104	1.100
	(0.892, 1.334)	(0.891, 1.334)	(0.902, 1.350)	(0.899, 1.345)
Years of schooling	0.987	0.988	0.988	0.987
	(0.949, 1.025)	(0.950, 1.026)	(0.951, 1.027)	(0.949, 1.025)
Marital status				
Unmarried	Reference	Reference	Reference	Reference
Married	1.387 **	1.384 **	1.381 **	1.383 **
	(1.124, 1.711)	(1.122, 1.708)	(1.119, 1.705)	(1.121, 1.708)
Number of family members	0.965	0.966	0.968	0.968
	(0.914, 1.019)	(0.915, 1.020)	(0.916, 1.022)	(0.917, 1.022)
Region				
Middle China	Reference	Reference	Reference	Reference
Western China	1.132	1.154	1.403	0.950
	(0.911, 1.407)	(0.687, 1.940)	(0.742, 2.653)	(0.586, 1.543)
Eastern China	1.330 *	1.009	0.902	0.886
	(1.070,1.653)	(0.606, 1.680)	(0.466, 1.746)	(0.551, 1.426)
Government communication	1.004	1.002	1.004	1.004
	(1.000, 1.008)	(0.996, 1.008)	(1.000, 1.008)	(1.000, 1.008)
Government prevention and control	1.024 ***	1.024 ***	1.024 ***	1.024 ***
	(1.018, 1.031)	(1.018, 1.031)	(1.015, 1.032)	(1.018, 1.031)
Government rescue	1.009 ***	1.009 ***	1.009 ***	1.007 *
	(1.006, 1.013)	(1.006, 1.013)	(1.006, 1.013)	(1.001, 1.012)
Government communication × Western China		1.000		
		(0.992, 1.007)		
Government communication × Eastern China		1.004		
		(0.997, 1.011)		
Government prevention and control × Western China			0.996	
			(0.987, 1.006)	
Government prevention and control × Eastern China			1.006	
			(0.997, 1.016)	
Government rescue × Western China				1.003
				(0.996, 1.009)
Government rescue × Eastern China				1.006
				(1.000, 1.013)
*N*	3837	3837	3837	3837
pseudo *R*^2^	0.145	0.146	0.146	0.146

95% CI in parentheses; * *p* < 0.05, ** *p* < 0.01, *** *p* < 0.001.

**Table 6:** Ordinary least squares model predicting risk perception and logit model predicting the public’s adoption of protective action recommendations (PARs).

Variables	Model 1	Model 2
	OLS	Logit
Gender		
Male	Reference	Reference
Female	9.290 ***	0.996
	(7.098, 11.482)	(0.830, 1.194)
Age group(years)		
<30	Reference	Reference
30–60	15.800 ***	1.193
	(12.423, 19.176)	(0.851, 1.672)
>60	−20.114 ***	1.057
	(−28.412, −11.815)	(0.582, 1.918)
Household registration		
Rural household	Reference	Reference
Urban household	−12.509 ***	1.486 ***
	(−15.094, −9.924)	(1.199, 1.841)
Years of schooling	0.808 ***	0.965
	(0.354, 1.262)	(0.926, 1.006)
Marital status		
Unmarried	Reference	Reference
Married	12.686 ***	1.122
	(10.018, 15.354)	(0.902, 1.397)
Number of family members	−2.454 ***	1.023
	(−3.160, −1.749)	(0.967, 1.082)
Region		
Middle China	Reference	Reference
Western China	−7.248 ***	1.339 *
	(−10.079, −4.417)	(1.068, 1.679)
Eastern China	2.106	1.295 *
	(−0.641,4.853)	(1.033,1.623)
Government communication	0.090 ***	1.002
	(0.040, 0.139)	(0.997, 1.007)
Government prevention and control	0.458 ***	1.014 ***
	(0.374, 0.542)	(1.007, 1.022)
Government rescue	0.190 ***	1.008 ***
	(0.138, 0.243)	(1.004, 1.014)
Risk perception		1.020 ***
		(1.018, 1.023)
*N*	3837	3837
adj. *R*^2^/pseudo *R*^2^	0.315	0.210

95% CI in parentheses; * *p* < 0.05, ** *p* < 0.01, *** *p* < 0.001.

**Table 7:** Bootstrap estimation of mediation effects.

Variables	Total Effect	Direct Effect	Indirect Effect	95% CIs of Indirect Effect	
				Lower Bound	Upper Bound
government communication → risk perception → adoption of PARs	0.039 *	0.020	0.019 ***	0.009	0.029
government prevention and control → risk perception → adoption of PARs	0.236 ***	0.161 ***	0.075 ***	0.061	0.095
government rescue → risk perception → adoption of PARs	0.124 ***	0.081 ***	0.043 ***	0.030	0.055

* *p* < 0.05, ** *p* < 0.01, *** *p* < 0.001.

## Appendix A

**Table A1:** The distribution of the sample.

Province	Population Size			Sample Size		
	Total	Male	Female	Total	Male	Female
Beijing	19,612,368	10,126,430	9,485,938	59	30	29
Tianjing	12,938,693	6,907,091	6,031,602	39	21	18
Hebei	71,854,210	36,430,286	35,423,924	216	109	107
Shanxi	35,712,101	18,338,760	17,373,341	107	55	52
Inner Mongolia	24,706,291	12,838,243	11,868,048	74	39	35
Liaoning	43,746,323	22,147,745	21,598,578	131	66	65
Jilin	27,452,815	13,907,218	13,545,597	82	42	40
Heilongjiang	38,313,991	19,426,106	18,887,885	115	58	57
Shanghai	23,019,196	11,854,916	11,164,280	69	36	33
Jiangsu	78,660,941	39,626,707	39,034,234	236	119	117
Zhejiang	54,426,891	27,965,641	26,461,250	163	84	79
Anhui	59,500,468	30,245,513	29,254,955	179	91	88
Fujian	36,894,217	18,981,054	17,913,163	111	57	54
Jiangxi	44,567,797	23,003,521	21,564,276	134	69	65
Shandong	95,792,719	48,446,944	47,345,775	287	145	142
Henan	94,029,939	47,493,063	46,536,876	282	143	140
Hubei	57,237,727	29,391,247	27,846,480	172	88	84
Hunan	65,700,762	33,776,459	31,924,303	197	101	96
Guangdong	104,320,459	54,400,538	49,919,921	313	163	150
Guangxi	46,023,761	23,924,704	22,099,057	138	72	66
Hainan	8,671,485	4,592,283	4,079,202	26	14	12
Chongqing	28,846,170	14,608,870	14,237,300	87	44	43
Sichuan	80,417,528	40,827,834	39,589,694	241	123	118
Guizhou	34,748,556	17,905,471	16,843,085	104	54	50
Yunnan	45,966,766	23,856,696	22,110,070	138	72	66
Tibet	3,002,165	1,542,652	1,459,513	9	5	4
Shaanxi	37,327,379	19,287,575	18,039,804	112	58	54
Gansu	25,575,263	13,064,193	12,511,070	77	39	38
Qinghai	5,626,723	2,913,793	2,712,930	17	9	8
Ningxia	6,301,350	3,227,404	3,073,946	19	10	9
Xinjiang	21,815,815	11,270,147	10,545,668	65	34	31
Total	1,332,810,869	682,329,104	65,048,1765	4000	2050	1950

**Table A2:** One-way ANOVA results: Differences between Western China, Middle China, and Eastern China.

Variables	Western China M (SD)	Middle China M (SD)	Eastern China M (SD)	*F*	*p*
Adoption of PARs	0.779 (0.414)	0.775 (0.423)	0.822 (0.382)	6.91	0.001
Government communication	70,783 (30,327)	75,909 (28,785)	73,692 (31,254)	8.60	0.000
Government prevention and control	70,334 (22,652)	75,014 (22,283)	73,805 (21,636)	14.82	0.000
Government rescue	74,104 (29,070)	74,405 (32,114)	79,058 (29,722)	11.56	0.000
Risk perception	53,347 (42,822)	61,887 (40,142)	66,131 (39,228)	34.78	0.000
